# Negotiating Unsustainable Food Transformations: Development, Middle Classes and Everyday Food Practices in Vietnam

**DOI:** 10.1057/s41287-021-00429-6

**Published:** 2021-07-07

**Authors:** Arve Hansen

**Affiliations:** Centre for Development and the Environment, University of Oslo, Blindern, P.O. Box 1116, 0317 Oslo, Norway

**Keywords:** Food, Vietnam, Malnutrition, Food environments, Middle classes, Consumption

## Abstract

Amidst calls for making food systems more sustainable, new unsustainable food transformations unfold alongside economic development. Explanations for unsustainable food transformations in emerging economies vary greatly, but there is widespread agreement that demand from new middle classes play a crucial role. Yet this demand is to a large extent co-created by systems of provision, and middle-class consumers are constantly navigating food transformations in a search for healthy and safe food. Focusing on Vietnam’s dramatic food transformations, and combining attention to the political economy of food with a social practice approach to consumption, the paper zooms in on the how middle-class households in Hanoi negotiate the rapid transformations of food systems and food environments. The paper concludes that new thinking on sustainable food systems is urgently needed and argues that vital insights can be gained by studying food practices and their interaction with everyday geographies of consumption.

## Résumé

Dans un contexte d’appels à rendre les systèmes alimentaires plus pérennes, de nouvelles transformations alimentaires non pérennes accompagnent le développement économique. Les facteurs qui expliquent ces transformations alimentaires non pérennes dans les économies émergentes varient considérablement, mais il est largement admis que la demande des nouvelles classes moyennes joue un rôle crucial. Pourtant, cette demande est en grande partie co-créée par les systèmes d'approvisionnement, et les consommateurs de la classe moyenne évoluent constamment sur le sujet des transformations alimentaires, à la recherche d'une alimentation saine et sûre. Cet article porte son attention sur les transformations alimentaires spectaculaires du Vietnam et combine un regard sur l’économie politique de l'alimentation avec une approche de la consommation par la pratique sociale. Il se concentre ainsi sur la façon dont les ménages de la classe moyenne à Hanoï approchent les transformations rapides des systèmes alimentaires et des environnements alimentaires. L'article en conclut qu'une réflexion renouvelée sur les systèmes alimentaires pérennes est nécessaire et urgente, et affirme que des informations vitales peuvent être obtenues en étudiant les pratiques alimentaires et leur interaction avec les géographies quotidiennes de la consommation.

## Introduction

That the world is facing a dramatic ‘triple crisis of malnutrition’ has been well established, with undernutrition, micro-nutrient deficiencies and overnutrition co-existing, often within the same country. While hunger and undernutrition remain far from eliminated despite decades of targeted effort, overnutrition is increasing at a much faster pace. More people are now overweight than underweight globally, and overweight and obesity continue increasing in all the world’s regions (FAO et al. 2019). The fact that low- and middle-income countries are also facing significant overnutrition challenges, combined with the environmental impacts of changing diets (Poore and Nemecek 2018), has led to a necessary broadening of the focus on food in global development and sustainability agendas. While undernutrition, poverty and food supply remain the main focus, all forms of malnutrition are now discussed as sustainable development challenges (FAO et al. 2018, 2019; World Bank 2019; Swinburn et al. 2019; Reisch and Gwozdz 2019). Based on a close reading of the many targets of the Sustainable Development Goals, Reisch and Gwozdz (2019) indeed conclude that healthy diets and healthy lifestyles now represent a pillar of sustainable development. The most recent Global Nutrition Report (2018) supports this view, and finds that malnutrition, including overweight and obesity, is a social and economic problem ‘holding back development across the world with unacceptable human consequences’ (p. 21) and that ‘progress to tackle all forms of malnutrition remains unacceptably slow’ (p. 29). Increasingly, food safety is also recognised as a fundamental development issue (Grace 2015), as well as a necessary component for achieving most of the SDGs (Grace 2017).

Although more food is produced in the world than ever before, the global food system is frequently portrayed as ‘broken’ (e.g. Carrington 2018) or at least as ‘failing’ (see Bene et al. 2019). The UN Environment Programme (UNEP) has called for a ‘food systems revolution’ (UNEP 2019a) and a ‘great food transformation’ (UNEP 2019b). However, while food transformations are indeed taking place in many parts of the world, they are often moving food systems in less rather than more sustainable directions. Large parts of the world are seeing transformations towards unsustainable and unhealthy diets consisting of more meat, more fast food, more ultra-processed food, and more unhealthy fat and sugar (Popkin 2017; Popkin et al. 2012). According to a recent Lancet Report (Swinburn et al. 2019), we are now facing a ‘global syndemic of obesity, undernutrition and climate change’. Despite pockets of improvement in health and nutrition, animal welfare and environmental protection, the global picture of food is bleak, and current global food systems are considered unsustainable whether we focus on health, the environment, human rights or socio-economic development (Lang 2014).

The potential benefits and positive spill-over effects of more sustainable and healthy diets have started to attract more attention in development research (Lang 2014; Reisch and Gwozdz 2019). There are enormous potential sustainability benefits from changing diets, mainly from reducing the consumption of animal products. Indeed, based on a comprehensive study of the environmental impacts of food production and consumption, Poore and Nemecek (2018, p. 991) argue that ‘dietary change can deliver environmental benefits on a scale not achievable by producers’. This potential obviously grows further if we factor in the ongoing and expected future ‘nutrition transitions’ in large parts of the world (Popkin et al. 2012). Indeed, the rapid economic growth and changing composition of food consumption in ‘emerging economies’ is expected to change the global food landscape the coming decades (Gandhi and Zhou 2014). In fact, studies find that consumption of highly processed food—the consumption of which is associated with increased risk of obesity and overweight (Holmes et al. 2018) is growing faster in low- and middle-income countries than in high-income countries (Moodie et al. 2013; Stuckler et al. 2012).

Explanations for unsustainable food transformations in emerging and developing economies vary greatly, but tend to focus on either the political economy of food provisioning or on the consumption patterns of new middle classes. It is widely accepted that unsustainable food transformations are closely connected to processes of economic development and urbanization, although the explanatory value of such macro-scale processes can be questioned (Stuckler et al. 2012). Scholars have pointed towards the ‘corporate food regime’ (McMichael 2013), the spreading of the ‘industrial grain-livestock complex’ (Weis 2007, 2013) or capitalism’s dependence on ‘cheap food’ (Moore 2015) as central, where the ‘world food economy’ sees increasing concentration of power over food (Clapp 2016), and capitalist logics favour industrialised agriculture and fast and highly processed food, to the detriment of people, animals and the environment (Weis 2013). On the demand side, both in development and nutrition research, much attention is given to the changing diets of new middle classes in emerging economies. While the role of these middle classes in development remain debated (see Birdsall 2015; Melber 2015), their explosive growth (Kharas 2017) and the fact that they are the ‘proclaimed bearers of the so-called development torch’ (Melber 2015, p. 248) place them at the core of sustainable development. When it comes to sustainability, they are seen as both culprits and heroes, through driving consumption booms while representing a hope for more sustainable consumption patterns (Wiemann 2015; see also Hansen 2020). In research on nutrition and development, they are usually portrayed as a driving force for unhealthy food transformations, due to demand for modern food spaces (Khonjo and Qaim 2019), as well as import-oriented diets and a general ‘global orientation’ (Pingali 2007). The latter relates to an asserted global convergence towards more ‘Western’ diets known to be unbalanced, high on fat, sugar and animal-source food (Popkin et al. 2012; Kearney 2010).

Despite the increased attention given to middle classes, there has been a lack of research on how the new middle classes ‘negotiate’ food transformations in everyday life. Indeed, beyond nutritional shifts, little attention has been given to the complexity of food consumption, including ‘how people interact with food sources to acquire foods as part of daily life’ (Turner et al. 2018, p. 93). In Asia and the Pacific, the region at the very core of global food transformations, a recent review by Montefrio and Wilk (2020) found that food consumption has attracted little attention, with the exception of consumer motivation studies within marketing and the behavioural and economic sciences (although see for example contributions in Sahakian et al. 2016; Ehlert and Faltmann 2019a, b). As I return to below, to the extent that consumption emerges in the global sustainability agenda overall, it tends to be through more or less rational consumers who simply need information to consume more sustainably. This despite the fact that decades of research within the broad field of consumption studies has shown that this simplification is unhelpful as a starting point for understanding consumption (see Evans 2019 or Warde 2017 for recent overviews). This field has seen a clear turn towards theories of practice in recent decades, which also represents the starting point of this paper, in combination with a focus on the political economy and everyday geographies of food. Drawing on long-term fieldwork in Hanoi, I employ this theoretical framework to the case of Vietnam, a country home to one of the world’s fastest growing economies and fastest expanding middle classes, which finds itself in the middle of rapid food transformations. Focusing on shopping and eating practices, I pay particular attention to how middle-class consumers negotiate ongoing food transformations in search of clean and healthy food. After outlining the methods used in the research that this paper draws on, the paper goes on to present the theoretical framework employed in the analysis. I then zoom in on the political economy of food transformations in Vietnam and how these relate to food practices among middle-class households in Hanoi.

## Methods

The paper draws on extensive fieldwork in Hanoi, mainly over three periods in March to May and in October 2017 and in March to April 2018, as well as a brief follow-up in October 2019. During these periods I conducted interviews with food, livestock and food systems researchers and experts working in international organisations, government officials, representatives of state-owned and private enterprises in the food sector, restaurant owners, market vendors, specialty food producers, and farmers, as well as members of 25 middle-class households, about food systems and food practices. All interviews were semi-structured, but varied considerably in style and length depending on context and available time. Approximately, half of the interviews were conducted in English and the other half in Vietnamese with an assistant translator. The households were recruited through a combination of purposive and snowball sampling with the aim of recruiting a broad variety of households. The sample included members from the lower ends to the upper income ends of the heterogeneous middle-class category (see Hansen 2020), and of different age groups (the youngest was 26 and the oldest 65 years old). With one exception, the households consisted of more than one person, and while a couple of the youngest interviewees lived with their parents, most had established their own family. Two interviews were done with couples together, and in total 19 of the household interviewees were female.

The research combined interviews with an ethnographically inspired approach to food practices. This aligns with practice approaches’ emphasis on going beyond ‘sayings’ to also study ‘doings’ to understand practices (Halkier and Jensen 2011). Concretely, this food ethnography involved engaging directly in Hanoian everyday food practices, such as going to wet markets, minimarts and supermarkets, and eating at a wide variety of food spaces, particularly those frequented by middle classes. Going to these places, combined with living in Hanoi over longer periods, gave me the opportunity to engage in a large number of more informal food conversations that have also informed the research. Relatedly, I draw on 10 years of work in Vietnam in short and long periods, where food has always represented a core interest. The research is thus also informed by a very large number of discussions about food and food systems, frequent lunches and dinner meals at office canteens and at the homes of people in several parts of the country, and an estimated couple of thousand restaurant and street food meals all over Vietnam.

## Studying Development, Consumption and Food Transformations

Although nutrition obviously implies consumption, as noted above, consumption is rarely the main focus in studies of development and malnutrition. For example in food system approaches, as pointed out by Mergenthaler et al. (2009, p. 426), ‘in spite of the hypothesized importance of both supply and demand side factors in the food system transformation, most of the available studies concentrate primarily on aspects of supply’. And, as stated above, when demand is included, it takes form as quantitative analyses of consumer choices, rather than as in-depth studies of food practices.

Despite increasing theoretical complexity in the field of development research overall, including on poverty and food, the rational consumer of neoclassical economics has usually gone unchallenged when the sustainability agenda deals with relative affluence. At least there appears to be a strong belief in the power of information in shaping individual choices. In other words, there is an assumption that consumers will consume healthier and more sustainable if provided the right information. A recent example is Poore and Nemecek’s (2018) account of the environmental impacts of food, where after several useful suggestions on how to design policies targeting producers, and after locating changing diets from below as the most promising venture for sustainable change, the suggested consumer policy aims at information alone. Even in the growing literature on ‘sustainable diets’, which argues for concentrating ‘policy attention on transforming consumption, not just production and distribution’ (Lang 2014, p. 240), information and dietary guidelines are core policy recommendations (Lang 2014). This is also the case in the world of sustainability policy, Shove (2014, p. 415) argues, where ‘efforts to nudge behaviour, modify attitude and encourage individuals to make better, greener choices’ dominate.

Providing information to consumers is both important and challenging, and involves regulating the power of commercial actors. However, both theory and practice in the field of sustainable consumption quite clearly shows that information alone is insufficient to change consumption patterns (see for example Shove 2014). In order to deal with unsustainable food consumption, a holistic approach that acknowledges the complexity of consumption is necessary. From the side of research, this includes in-depth studies of food in everyday life, including urban geographies of food. Furthermore, I argue, it requires studying the relationship between food practices and the political economy of food.

### Theoretical Approach: Political Economy Meets Food Environments and Food Practices

The frontier of research on consumption has for more than a decade been dominated by theories of practice and a focus on mundane consumption, everyday life and the complex and gradual processes involved in normalising new forms (and ever-higher levels) of consumption (see Evans 2019). This paper is influenced by the ‘practice turn’ in consumption research, seeing consumption as taking place through shared social practices. Studying practices means studying social phenomena between individuals and social structures, in between *homo oeconomicus* and *homo sociologicus* (Dubuisson-Quellier and Gojard 2016). Simply put, from a practice approach, consumption is understood not mainly as an outcome of individual choices but through its position in the many parts and activities that make up everyday life. Food is a case in point. While people certainly make food choices, and while it certainly is both possible and common to express status or identity through food, most of our food consumption can more accurately be characterised by ‘repetition, routine and convention’ (Warde 2016). Food practices consist of acquiring, storing, cooking, eating and discarding food, and are clearly shaped by social and material contexts, as well as ‘cultural notions of appropriateness’ (Paddock 2017, p. 135).

Food practices are part of, and co-shaped by, an intricate web of other social practices—related to mobility, the home, offices and more—that make up practice ‘bundles’ and ‘complexes’ (Shove et al. 2012). Furthermore, the ‘systems of provision’ (Fine 2013) of food combined with infrastructure and urban geography strongly influence food consumption patterns. These relations are, as Shove et al. (2018, p. 210) remind us, ‘shaped by the ambitions and actions of states, companies, citizens and consumers’. Indeed, demand is partly both made and steered by policy and commercial actors (Rinkinen et al. 2021). While noting these connections, however, the ‘practice turn’ has had less to say about political economy (Welch and Warde 2015). It has also had very little to say about the impact of development processes on everyday practices, since research to a large extent has focused on affluent societies. In order to connect food practices with the political-economic and urban-geographical contexts they take place in, I put emphasis on how changing ‘food environments’, (FAO et al. 2019; Turner et al. 2018)—simply put where people acquire their food—co-shape food practices. Some of these effects are obvious. If the local wet market is closed, or rendered unsafe, people will use other stores. Others are less obvious, like the long-term effects of marketing or the ways supermarkets are spatially structured to affect the consumption of certain kinds of food. Food practices are in turn shaped by other material factors, such as transport mode and household appliances like refrigerators (Rinkinen et al. 2019). They are furthermore shaped by social contexts, such as norms, opinions amongst friends and socially ‘established understandings of what courses of action are not inappropriate’ (Warde 2005, p. 140). What this means is basically that consumers have motivations and make choices, but these choices are fundamentally influenced by their positions as social subjects, as well as shaped and narrowed down by social and material surroundings. Options are often also significantly constrained by the power of corporations to determine what food consumers can access (Wilk 2018). In the vocabulary of theories of practice, this relationship between structures and the agency of individuals can be understood as ‘bounded creativity’ (Nicolini 2012). It is this space for individual creativity within structural constraint I in this paper approach as negotiations, which I in turn use to analyse the strategies my informants in Hanoi employ in the encounter with changing food systems and food environments.

## Food Practices and the Political Economy of Urban Food Transformations in Vietnam

A key part of Vietnam’s ‘development success story’ (e.g. Baum 2020) has been poverty reduction (Banik and Hansen 2016), as well as an associated rapid improvement in nutrition (Marzin and Michaud 2016). Food production is a clear example of the success of *doi moi*, as it saw Vietnam develop from food scarcity and import dependence to a major agricultural exporter (Marzin and Michaud 2016). Indeed, the World Bank considers Vietnam a ‘food security success’ that many countries are trying to learn from. Coupled with increasing imports, the increase in domestic agricultural production has made possible increases in calorie intake. From 1986, the year the reforms were initiated, until 2013, daily per capita calorie intake increased 33%, mainly due to an increase in calories from animal products (Hansen 2018). Still, however, about 11% of the population are considered undernourished and 2% ‘severely food insecure’ (FAO et al. 2018), and the poorer mountainous regions and ethnic minorities fare far worse off than the rest of the population (Raneri et al 2019, p. 4). And while a more diversified diet has helped lower the prevalence of undernutrition, Vietnam has simultaneously been seeing food transformation towards higher consumption of salt, ultra-processed food and soft drinks, and in recent decades a decline in consumption of fresh fruit, vegetables and seafood (Raneri et al. 2019). While such dietary changes and nutritional shifts have often been described as demand driven, food practices have changed through changes in ‘supra-practice configurations’ (Welch and Warde 2015) as part of development processes. In particular, they have changed as part of changes in the political economy of food, which have involved often dramatic changes in food environments (see Hansen and Jakobsen 2020a, b; Wertheim-Heck 2015). As theory would predict, Vietnam has over the recent decades seen an increase in overweight and obesity, including among children, as the country is facing a crisis of malnutrition (Loan Minh Do et al. 2016). These trends are expected to continue alongside a predicted rapid urbanisation in coming years as well (World Bank 2016).

Health was a vital concern to my middle-class interviewees in Hanoi. A woman in her early thirties, herself a recently converted vegetarian, explained how there had been changes in how Hanoians valued different kinds of food and that now people ‘really just want to eat what’s good for their health’. (Interview, March 2017). Another woman of the same age described what she saw as a deeply psychological worry about hunger in her parents’ generation, and explained that her parents would always complain that she was too skinny and should eat more (Interview, March 2017). This echoes Ehlert’s (2019) findings on how the valuation of the ‘chubby child’ is deeply connected to the notion of a good mum able to feed her children. Similar stories are common regarding meat, despite the rapid ‘meatification’ of diets seen in recent decades. Indeed, meat still in many ways symbolises progress and is widely seen as healthy and a core ingredient for strength (Hansen 2018). But several of my informants, including the vegetarian woman introduced above, claimed that the long-held view that nothing is better for your health than meat was being challenged by more knowledge about the health consequences of meat. Indeed, more meat intensive and fat and sugar intensive food practices coexist with attempts to cut back on meat consumption among many consumers (Hansen, forthcoming), as well as widespread worry about the negative health impacts of nutritional changes, both among consumers and the authorities. While such worries do not automatically lead to changes in food practices, and while there indeed often is a sharp contrast public obesity discourses and popular ‘truths’ about body shape and health (Ehlert 2019), they do clearly underline the point that middle class consumers are just as much ‘victims’ as ‘drivers’ in unsustainable food transformations.

Food environments in Hanoi have changed rapidly over the past decades, and many of my interviewees worried about the impacts of new food environments on their children’s health. With its rapid growth and expanding middle class, Vietnam has unsurprisingly become home to many of the large global and regional fast food chains. KFC has been there since 1997, Burger King came in 2012, McDonalds in 2014, while Jollibee from the Philippines and Lotteria from South Korea opened their first Vietnamese branches in 1996 and 1998 respectively. Fast food has seen a boom in Vietnam in the recent decade and is growing in popularity (Hansen and Jakobsen 2020a, b). That said, sales have not lived up to expectations for many of these foreign giants. KFC and Lotteria are widespread, and Jollibee growing, but McDonald’s have fallen far short of their targets and Burger King has even been closing outlets in recent years (Saigoneer 2016, 2017). The success recipe, it seems, is to adapt to local food practices and serve rice lunches at a fairly low price (Hansen, forthcoming).

This may be changing, and my older interviewees identified a generational shift. A common perception is that while older generations prefer traditional Vietnamese cuisine, children have fully embraced foreign foods, including fast food. A few of my interviewees had children they referred to as overweight. A mother of two in her late thirties was one of them. As she put it, ‘children like fast food, but it makes them overweight’ (Interview, May 2017, translated from Vietnamese). Her worry extended to overconsumption of sugar and to instant noodles, which she described as not good for health due to low-quality ingredients. Several informants described similar worries, often referring to children and young people’s appetite for fast food. While ‘Western’ food is widely described as unfitting for Vietnamese taste buds, many children have grown up with these tastes. This could lead to more significant transformations in the future. Pierre Bourdieu (1984, p. 71), among the most important practice and consumption theorists, considered tastes of food as where ‘one would find the strongest and most indelible mark of infant learning’. And in Hanoi it is common to take children for fast food meals as a treat, even among many who are skeptical of the health impacts of such food.

The fast food enterprises may be given a helping hand by authorities in Vietnam. A popular perception is that foreign fast food cannot compete with street kitchens (see Saigoneer 2017), which are considerably more widespread and popular than ‘modern’ fast food restaurants. The local street food scene often consists of high quality slow food served even faster than foreign fast food, often at a fifth of the price. Eating at these is deeply embedded in everyday lives of large parts of Hanoians. And although also undergoing certain ‘meatification’ (Hansen 2018), they represent an important source for quick, affordable, nutritious and healthy food. Yet, street food is under threat by Hanoi city authorities’ ambitions to develop a modern, prosperous and ‘civilized’ city. On the one hand street kitchens have suffered from the ‘Clean Up the Sidewalks Campaign’ implemented in 2017 (see Turner and Ngo (2018), and on the other they are, alongside wet markets (Atomei 2017), seen as both old-fashioned, unruly and unhygienic. The latter point is unsurprising, as the existence of such largely informal sectors of the food system is known as a main cause of food safety challenges in low and middle income countries (Grace 2015).

While street food remains important and highly popular, the worry about food safety is shared by many consumers, and intensified by the food scares discussed below. Again, my interviewees had different ways of coping with and negotiating this. Some, particularly among the wealthiest, would avoid all forms of street kitchens, others would only eat at these when alone and would try to limit the exposure of children to street food. Many would state that they were uncertain about the safety, but most would continue eating street food as a central part of everyday food practices. Many of the households I interviewed would eat outside every single day, some even two or three meals a day. That would often include *pho* or other noodle dishes in the morning, and rice meals during lunch. The convenience, affordability and relatively high quality of street food enable and strengthen its position in everyday practice bundles. Yet the future is uncertain, and the sceptical position of authorities and some consumers coupled with a tendency towards popular street food dishes being co-opted by larger commercial actors who move eating practices indoors to air-conditioned spaces make future configurations to these practices likely. This is certainly also the case for food shopping practices, where traditional markets is facing intensified competition.

### Food Strategies: Traditional Markets, Supermarketisation and New Provisioning Networks

Vietnam has seen a range of food scares and cases of food contamination over recent years (see Ehlert and Faltmann 2019a, b), which have led to widespread worries across the country about what is healthy and safe to eat, who among retailers and producers can be trusted in delivering safe food, as well what kind of information about food safety is reliable. Food anxiety is indeed a defining part of contemporary urban Vietnam (Ehlert and Faltmann 2019a, b), strengthened by a strong media focus on dubious agricultural and street retail practices. Although the extent of food contamination remains uncertain (Nguyen-Viet et al. 2017), food safety is by now a well-recognized dilemma by consumers, and influences what they purchase and where they do their shopping for food (Raneri and Wertheim-Heck 2019). Food concerns like these are far from unique to Vietnam, although the rapidity of changes in systems of provision for food and the context of an authoritarian state where people already have little trust in information adds complexity to the situation. Still, consumers do what they can to cope and find ways of managing uncertainty.

Shopping for food in Hanoi has over generations been built around the traditional wet market and these represent crucial food institutions in the city (see Atomei 2017). Typically, food practices built around wet markets include shopping for fresh food every day, often early in the morning. Most of the households I interviewed would however report worries about the quality of food in markets, and had developed different strategies to determine what is safe to eat. These strategies included avoiding Chinese produce,^1^ using ‘trusted vendors’ and using one’s senses and personal expertise to determine whether produce is safe to eat (see also Faltmann 2019; Figuié et al. 2019). As explained by a middle-class single mother in her late twenties:

I can tell which one is fake. Which one actually comes from the countryside, and which one does not […] Through experience I learn how to tell.

(Interview, Hanoi, March 2017)

She would also use vendors that were trusted to be speaking the truth about the origin of their products, often claiming to acquire products directly from rural areas. Food system experts I talked to doubted that this was possible, since the main source of products are large whole-sale markets, whose vendors in turn tend to be uncertain about the origins (personal communication, Hanoi, October 2019). This is nevertheless a popular way of negotiating uncertainty.

Some of my more affluent interviewees shook their heads at these common strategies for acquiring safe yet affordable food. Others would refer to the lack of these competences, as well as competences in negotiating price, as a reason for going to the supermarket instead, or they considered going to the market as something their mothers, who had a lot more time on their hands, would do. In other words, more harried everyday lives and changes in other practices, related to school, work, and caring for children, disallowed such food practices. Still, for large parts of the Hanoian population, wet markets represent a crucial venue for food shopping. This is the case for the middle classes, but is even more crucial for poorer segments of the population (Wertheim-Heck et al. 2019). Nevertheless, traditional wet markets are facing challenges from several directions at once. Both city and national authorities are supporting a shift towards ‘modern’ food outlets and generally seem to consider wet markets outdated in aims to modernize the city (Atomei 2017; Wertheim-Heck 2015). This tendency was intensified during the strict measures to stop the Covid-19 outbreak during spring 2020 (Wertheim-Heck 2020). Meanwhile, new supply chains are developing around supermarkets, and many consumers are shying away from traditional markets due to worries about the safety of market products, paving the way for significant changes in food systems and urban food environments.

As is common in middle-income countries around the world (Stuckler et al 2012), multi-national food companies and supermarket chains are making a strong presence in Hanoi. Although among the slow starters in the region (Reardon and Timmer 2007), ‘supermarketisation’ has taken off in Vietnam, with government support (Wertheim-Heck et al. 2019). Although the vast majority of food purchases still take place in traditional wet markets, according to World Bank (2016) estimates sales from hypermarkets, supermarkets and convenience stores doubled between 2005 and 2013, reaching an estimated 15% of total food sales in the country. This sector is also seeing increasing investments from domestic and foreign actors (World Bank 2016), with the VinGroup the most powerful domestic corporation with a very rapid expansion of small and large VinMart outlets, and the Thai-owned Metro (TCC Holding) and Big C (Central Group), Korean LOTTE Mart (LOTTE Group) and Japanese AEON among the largest foreign actors. This is an important development, and represents significant changes in food practices and supply chains. Supermarketisation is a good example of what Shove (2003, p. 9) has located as critical avenues for consumption research: ‘the big, and in some cases, global swing of ordinary, routinized and taken-for granted practice’. Crucially, supermarket shopping is generally known to have strong effects on the type of food consumed, particularly towards more consumption of highly processed food (see Boysen et al. 2019; Demmler et al. 2018).

While supermarket chains obviously respond to potential demand, they are also part of co-creating demand (Qaim 2017). In Hanoi, it is hard to estimate to what extent consumers have demanded supermarkets, but they have certainly not automatically shifted their shopping practices to these. Instead of consumers changing towards supermarkets overnight, supermarkets become gradually integrated into food practices, starting with particular food items, mainly processed food. Supermarkets in Hanoi are often used in combination with traditional outlets, similar to what Khonje and Qaim (2019) report from several African countries. Practices are ‘sticky’ and habituated consumption patterns usually change very slowly (Warde 2016). However, worries about food safety had clearly made its way into food practices and pushed many consumers towards supermarkets.

This trend was obvious among my interviewees, although several of them continued using traditional markets for most of their food shopping. A man in his late twenties who lived in a shared apartment, said that just a few years ago, he would go to local markets to buy food. But due to the many incidents of food contamination, he started going to convenience stores instead (Interview, April 2017). Similarly, a retired, upper-middle income woman living in an old villa in Hanoi’s French Quarter, had used traditional markets her whole life, but told me she recently changed to going only to supermarkets. In the process, she had changed from shopping for food every morning to going once every week. She would however only go to the Japanese up-scale chain Aeon. She explained how her trust was in many ways related to the company’s size and reputation, as well as price:

Researcher: So you don’t buy food at the market anymore?

Interviewee: Almost none. Because we don’t know where it comes from. I also choose the supermarket. There are some supermarkets that may not have strict regulation, so I don’t choose them.

[…]

Researcher: Is the food [at Aeon] imported from Japan?

Interviewee: No. They buy it here [in Vietnam], but the quality is better. And they have a reputation, so they have to protect their reputation by having good quality food. They are so big, so if they have an incident it will be bad for business.

Researcher: How often do you go food shopping?

Interviewee: Aeon is very far away. It costs 100.000 [VND] for a small car from here to Aeon. So I go once per week. Lotte is a little bit expensive, and they don’t have a lot of products. Because the location is right in the middle [of the city], it is more expensive. Big C is another huge one. It’s really cheap. The cheapest, but I worry. Because the prices are so low, so what about the quality? It may be dangerous. Big C is aiming for people with lower income.

(Interview, Hanoi, March 2017, translated from Vietnamese).

The logic that the largest companies must protect their reputation and thus can be trusted is interesting, and indeed something I have heard often in Hanoi. The level of trust of supermarket chains among consumers varies greatly. In my household interviews, like the one above, as well as in informal food conversations, chains like Big C from Thailand were often mentioned as unsafe and catering to ‘poorer’ consumers, whereas Vietnamese VinMart, Japanese Aeon and Korean Lottemart were often mentioned as safe. Word of mouth and social media seemed to represent central sources for judgements about food safety, although Japanese and Korean chains obviously also benefit from their respective countries of origin’s overall positive reputation in Vietnam.

The few large supermarkets in Hanoi are mostly placed outside the city centre, and thus, like the above quote shows, represent a break with common food practices. The situation for convenience stores is different, and their number have increased at a very rapid pace. Ten years ago there were very few minimarts in Hanoi, while now there is at least one in every neighborhood. Indeed, Hanoi authorities aimed to have 1,000 convenience stores in place by 2020 (Vietnam Investment Review 2019). Vingroup aimed to alone have 4000 such stores nationwide by 2020 (Vietnam Investment Review 2018). Alongside these new mini-marts, new ‘clean food stores’ have emerged. These are very popular, despite their often very high prices. Indeed, a quite well-off interviewee said she used these stores even though prices were often three times higher than at the market. She stated not trusting the market nor supermarkets, and would only use ‘clean’ and organic food stores (Interview, March 2017). While these are examples of the foodways of the rich and privileged, different versions of clean food stores are popular among broader segments of the middle classes.

Many households I interviewed also spoke of a desire to shop food directly ‘from the countryside’. The desire to get food directly from rural areas seems to be connected to certain urban romanticising of rural Vietnam, which stands in a rather paradoxical relationship to the prevalent deep distrust towards Vietnamese agriculture. There is, it seems, in the imaginaries of urban consumers, a ‘pure’ countryside out there, producing safe, clean and organic food the way Vietnamese food originally was produced. And in a situation where no one knows who to trust in the food system, the best bet is personal connections. This has developed into alternative food networks (see Kurfürst 2019), where relatives, neighbours or friends cooperate to acquire safe food. Others would purchase food online to the same ends. A young woman very engaged in issues of diets and food safety reported online food delivery as a common strategy for her. The food would be bought online and then delivered to Hanoi through regular bus services:

So, normally I also order online, some people […] know a good source and they [are] selling online from the rural area. And my family also, on my mum’s side, […] a lot of people there still do farming, so they reserve some for the family and they send to Hanoi for us. I know a lot of my colleagues and people around me do in the same way too. They know the source somewhere that they can trust. And they get food delivered from que, from the rural… Because they don’t trust the things they buy in the supermarket. Cause there’s a lot of pesticides and a lot of preservation and stuff. (Interview, March 2017).

These practices are largely undocumented but likely to be extensive. Neighbours often cooperate and order together, and also in areas with access to both traditional markets new supermarkets, many would supplement their food shopping by sourcing directly from farmers (see also Raneri et al 2019, p. 21). Some also do their own urban gardening of vegetables and herbs, often partly due to concerns for food safety (see also Kurfürst 2019).

In summary, middle-class households employ a wide range of strategies to try to acquire ‘clean’ food. The notion of clean (*xach*), refers mainly to health concerns, in other words to uncontaminated food. There is also a growing environmental awareness among consumers, particularly among younger segments, although this seems to still have negligible effects on food practices (see also Faltmann 2019). There is however a general sense of lack of information, combined with a widespread distrust of any kind of certification (see Faltmann 2019 for overview). The sentiment can be summarised by the following statement by an older interviewee, although she was specifically referring to market vendors:

*Even the people who go check the hygiene, they would take money under the table and give them the certification, so how do you know what to trust?* (Interview, Hanoi, March, 2017, translated from Vietnamese).

## Negotiating Unsustainable Food Transformations: Concluding Discussion

We know much about how food systems work, including what to expect from ongoing food transformations. Although playing out in different ways in different contexts, we can expect economic development to be accompanied by supermarketisation (Qaim 2017), the industrialisation of agriculture (Weis 2007; Clapp 2016), and changing consumption patterns towards more fat, more sugar and overall less healthy food (Popkin 2017). We know that we can expect spikes in obesity rates and non-communicable diseases due to such dietary changes (Popkin et al. 2012), and that the environmental footprint of food is likely to continue increasing dramatically (Poore and Nemceck 2018). These seem to be fundamental features of the ‘industrial diet’ (Winson 2013), with its deep inequalities in access to safe and healthy food (Otero et al. 2018). And we know that such transformations partly contribute to and are partly driven by, food safety challenges (Figuie et al. 2019). But we still do not have sufficient knowledge of how consumer navigate such transformations in and through everyday food practices, and how these navigations, or negotiations, can be built upon to make diets more healthy and sustainable as well as avoid unsustainable food transformations.

The literature on development, food systems change and nutrition transitions leaves an impression of a ‘natural’ processes towards unsustainable food systems alongside economic development. As people get richer and middle classes emerge, the story goes, they start demanding ‘modern’ food environments and start eating less healthy and less sustainable food (see Hansen 2018 for discussion). However, as pointed out in this paper, these are often planned developments. Like the case of Vietnam shows, Vietnamese authorities are in the name of progress systematically favouring exactly the kind of unsustainable and ‘obesogenic’ food systems and food environments that are blamed for the ‘global syndemic of obesity, undernutrition and climate change’ (Swinburn et al. 2019). Importantly, the findings presented in this paper show how middle-class households in Hanoi are trying to cope with the changes in Vietnam’s food system. First and foremost, they are anxious about the health effects of changing food systems and food environments and how to acquire clean and safe food and how to cope with a situation of deep distrust towards both producers and government regulation of the food sector. But the findings also show that middle classes do have agency in negotiating the changes and in carving out a variety of strategies and solutions, ranging from trying to limit the impact of foreign fast food chains on the children’s diets through using senses to determine the safety of vegetables to relying on alternative food networks. Importantly, however, this agency is bounded, and individual consumption patterns are fundamentally shaped by practices, food environments and the political economy of food. The normalization and naturalisation of unsustainable consumption patterns is indeed at the heart of global food transformations.

Political and economic structures strongly shape food practices. Yet research shows that there are significant challenges and limitations in dealing with unsustainable transformations from a production angle (Poore and Nemceck 2018). Thus, the clearest policy recommendation to extract from this paper is to take the complexity of consumption seriously. Thus, taking into account the ‘bounded creativity’ among emerging middle classes in acquiring clean and healthy food, new thinking is urgently needed on how to deal with transformations from the demand side. A consumption approach to food systems change that goes beyond the choices of individuals and embrace the complexity of food consumption can lead to new insights and open new avenues for research and policy towards sustainable food. By studying food practices and their interaction with food environments and the political economy of food, it is possible to learn from people dealing with food transformations in their everyday lives, particularly by studying how healthier and more environmentally friendly foodways can be supported as building blocks towards more sustainable transformations. To this end, much more research is needed on sustainable consumption and potential sustainability trajectories in emerging economies.

## Footnotes

1 Due to both deep anti-Chinese sentiments in Vietnam and to a common belief that Chinese agriculture uses excessive amounts of pesticides. The latter does have root in reality, as China is the world’s largest consumer of agricultural chemicals, according to Wu et al. (2018) using as much as 30% of the world's fertilizers and pesticides on 9% of global cropland.
